# Data on transnational ecological compensation under a ‘no net loss’ biodiversity policy

**DOI:** 10.1016/j.dib.2023.109324

**Published:** 2023-06-16

**Authors:** Anna Lou Abatayo, Joseph William Bull, Niels Strange

**Affiliations:** a Environmental Economics and Natural Resources Group, Wageningen University and Research, Hollandseweg 1, Wageningen 6706KN, the Netherlands; b Department of Biology, University of Oxford, 11A Mansfield Road, Oxford OX1 3SZ; c Department of Food and Resource Economics, University of Copenhagen, Rolighedsvej 23, 1958 Copenhagen, Denmark; d Center for Macroecology, Evolution and Climate, University of Copenhagen, Universitetsparken 15, Bld. 3, 2nd floor, DK-2100 Copenhagen

**Keywords:** Survey, Conservation, Spain, Denmark, Ghana

## Abstract

We conducted surveys in Denmark, Spain, and Ghana to solicit individual preferences for national and international ecological compensation for forest cover lost in the participant's home country due to the construction of a road. In the same survey, we also solicited individual socio-demographic characteristics and preferences, such as their gender, their risk preferences, whether they think individuals in Denmark, Spain, or Ghana can be trusted, etc. The data is useful for understanding individual preferences for national and international ecological compensation under a net outcomes type biodiversity policy (e.g., “no net loss”). It can also be used to understand how individual preferences and socio-demographic characteristics can be used to understand an individual's choice for ecological compensation.

**Specifications Table**

**Table:** 

Subject:	Economics, Ecology, Climate and Environmental Finance
Specific subject area:	Individual opinions for what constitute sufficient ecological compensation for forest habitat loss due to infrastructure development
Type of data:	Table
How the data were acquired:	The data was acquired through anonymous individual surveys in Denmark, Spain, and Ghana. The survey was originally written in English, translated to Danish and Spanish and re-translated back to English by a different translator. The English version of the survey is uploaded as a supplementary material of the related research article [1]. The survey was run manually (i.e., paper and pen). The results were separately encoded by two research assistants. The encoded results were compared with one another to check for accuracy.
Data format:	Raw
Description of data collection:	All participants participated in an economic experiment prior to answering the survey. Participants in Denmark and Spain were recruited through a database of university participants while participants in Ghana were recruited through in-class flyers and advertisements.
Data source location:	The surveys were conducted in three countries:Denmark • Institution: University of Copenhagen • City: Frederiksberg Spain • Institution: Pompeu Fabra University • City: Barcelona Ghana • Institution: University of Ghana • City: Accra
Data accessibility:	Repository name: Mendeley Data Data identification number: 10.17632/sm9t5s63rf.2 Direct URL to data: https://data.mendeley.com/datasets/sm9t5s63rf[2]
Related research article:	J.W. Bull, A.L. Abatayo, N. Strange, Counterintuitive proposals for trans-boundary ecological compensation under ‘no net loss’ biodiversity policy, *Ecological Economics*. 142 (2017): 185-13. https://doi.org/10.1016/j.ecolecon.2017.06.010. [1]

## Value of the Data

• The data is useful in understanding individual preferences for national and international ecological compensation.
• The data is useful for researchers, practitioners, and policy makers working on ecological compensation and net outcomes type biodiversity policy (e.g., “no net loss”, “biodiversity net gain”, “nature positive”).
• The data can shed light on how different socio-demographic characteristics and preferences can influence national and international ecological compensation.
• The data can be combined with other existing data on national and international ecological compensation.
• The data can be combined with another dataset on donations to bird conservation [3], published as another research article [4], to understand the relationship between preferences for ecological compensation and actual donations to bird conservation.

## 1 Objective

The objective of the dataset is to collect individual perceptions of what a suitable ecological compensation is for the loss of a forest habitat due to infrastructure development in their country. Participants were asked to specify how much ecological compensation should be in their country as well as in the two other countries (i.e., Denmark and Spain if the person is from Ghana) under different scenarios of forest cover trends in their country and the two other countries. The data allows the reproduction of all the statistical analysis and results of the original article, and hence, contributes to a more open science.

## 2 Data Description

A. Data Access

The data can be downloaded from Mendeley Data. To download, click on “Download All 59 KB”. Unzip the file and rename the unzipped folder as “Data”. Your “Data” folder should contain the following files:

(1) !ReadMe.txt
(2) ∼Codebook.txt
(3) 00 Runme.do
(4) 01 WideToLong.do
(5) 02 SumStat.do
(6) 03 tTest.do
(7) 04 Regression.do
(8) data_wide.dta
(9) data_wide.csv

The first step is to look at the “!ReadMe.txt” and the “∼Codebook.txt” files. The former contains information regarding the original article, the spatial and temporal coverage of the data, and instructions on how to reproduce the output of the original article. The latter is a data dictionary and contains detailed information, including frequency tables, of the variables in the data. The file “data_wide” is the dataset. It is provided in both “DTA” and “CSV” formats.

If you have Stata 13 MP/SE or higher installed, open the do-file “00 RunMe.do” and change line 57 to the directory that points where your “Data” folder is. For instance, if your “Data” folder is in your Apple desktop, change your directory to “/Users/username/Desktop”. To run all do-files, including the summary statistics and analyses for the related research article [1], run the entire “00 RunMe.do”.

Alternatively, if you don't have Stata 13 MP/SE installed, you can open the relevant data file, “data_wide.csv” your relevant statistical software. However, to run the rest the analyses for the original research article, you will need to first transform “data_wide.dta” from wide to long format. In Stata, the transformation is done using the do-file “01 WideToLong.dta”.

B. Data Overview

The data contains 59 variables and 691 observations, derived from a survey conducted in Denmark, Spain, and Ghana. Each observation is identified by the unique identifier, “uniqueid”. This variable is a concatenation of the following: “<country>” + “000” + “<session ID>” + “000” + “<subject ID>”. The string variable “country” determines which country an observation is from. All participants are nationals of the country they took the survey in (i.e., only Danes in Denmark, Spaniards in Spain, and Ghanaians in Ghana were allowed to participate). Session and Subject IDs are numbers from 1 to 20 and 1 to 12, respectively. These identifiers (i.e., country, session, and subject) can be used to merge this dataset with another dataset on bird conservation [3], [4].

Table 1 provides a list of variables and their descriptions when the data is in wide format. When transformed to long-format using the do-file “01 WideToLong.dta”, the variables d_*, s_*, and g_* are stacked together (i.e., d_like, s_like, g_like are stacked to form just one variable called “like” and the variables d_trust, s_trust, and g_trust are stacked to form just one variable called “trust”). The same holds for the all the variables f* (i.e., everything is stacked as one variable and new variables for the (1) case, (2) the country the ecological compensation is coming from, and (2) the country the ecological compensation is going to are created to differentiate the f* variables).

**Table 1:** Variable list with descriptions.

Variable Name	Storage Type	Display Format	Variable Label
uniqueid	str17	%17s	Unique ID
country	str7	%9s	Country
session	byte	%10.0g	Session ID
subjectid	byte	%10.0g	Subject ID Number in Session
age	byte	%10.0g	Age
gender	byte	%10.0g	Gender
student	byte	%10.0g	Are You a Student?
educ_attain	str23	%23s	Highest Educational Attainment
civilstatus	byte	%11.0g	Marital Status
children	byte	%10.0g	Number of Children
conserve_envi	byte	%16.0g	Conserves Environment?
birdlife	byte	%16.0g	Likes Birds?
risk	byte	%10.0g	Measure of Risk (0-risk averse, 10-risk loving)
d_like	byte	%16.0g	Like Danes?
d_trust	byte	%16.0g	Danes Can Be Trusted?
d_cooperate	byte	%16.0g	Danes Are Not Cooperative?
d_nature	byte	%16.0g	Danes Care for Nature?
d_bird	byte	%16.0g	Danes Do Not Protect Migratory Birds?
d_wealthy	byte	%16.0g	Danes are Wealthy?
s_like	byte	%16.0g	Like Spaniards?
s_trust	byte	%16.0g	Spaniards Can Be Trusted?
s_cooperate	byte	%16.0g	Spaniards Are Not Cooperative?
s_nature	byte	%16.0g	Spaniards Care for Nature?
s_bird	byte	%16.0g	Spaniards Do Not Protect Migratory Birds?
s_wealthy	byte	%16.0g	Spaniards are Wealthy?
g_like	byte	%16.0g	Like Ghanaians?
g_trust	byte	%16.0g	Ghanaians Can Be Trusted?
g_cooperate	byte	%16.0g	Ghanaians Are Not Cooperative?
g_nature	byte	%16.0g	Ghanaisn Care for Nature?
g_bird	byte	%16.0g	Ghanaians Do Not Protect Migratory Birds?
g_wealthy	byte	%16.0g	Ghana are Wealthy?
fcase1_denmark	double	%10.0g	Scenario 1: New Forest in Denmark
fcase1_spain	double	%10.0g	Scenario 1: New Forest in Spain
fcase1_ghana	double	%10.0g	Scenario 1: New Forest in Ghana
fcase2_denmark	double	%10.0g	Scenario 2: New Forest in Denmark
fcase2_spain	double	%10.0g	Scenario 2: New Forest in Spain
fcase2_ghana	double	%10.0g	Scenario 2: New Forest in Ghana
fcase3_denmark	double	%10.0g	Scenario 3: New Forest in Denmark
fcase3_spain	double	%10.0g	Scenario 3: New Forest in Spain
fcase3_ghana	double	%10.0g	Scenario 3: New Forest in Ghana
fblackcaps_denmark	double	%10.0g	Scenario 4: New Forest in Denmark
fblackcaps_spain	double	%10.0g	Scenario 4: New Forest in Spain
fblackcaps_ghana	double	%10.0g	Scenario 4: New Forest in Ghana
fieldcateg	float	%9.0g	Field of Study
case1sum	float	%9.0g	Scenario 1 Total
case2sum	float	%9.0g	Scenario 2 Total
case3sum	float	%9.0g	Scenario 3 Total
case4sum	float	%9.0g	Scenario 4 Total
pct1den	float	%9.0g	Scenario 1: Percentage Denmark
pct1spa	float	%9.0g	Scenario 1: Percentage Spain
pct1gha	float	%9.0g	Scenario 1: Percentage Ghana
pct2den	float	%9.0g	Scenario 2: Percentage Denmark
pct2spa	float	%9.0g	Scenario 2: Percentage Spain
pct2gha	float	%9.0g	Scenario 2: Percentage Ghana
pct3den	float	%9.0g	Scenario 3: Percentage Denmark
pct3spa	float	%9.0g	Scenario 3: Percentage Spain
pct3gha	float	%9.0g	Scenario 3: Percentage Ghana
pct4den	float	%9.0g	Scenario 4: Percentage Denmark
pct4spa	float	%9.0g	Scenario 4: Percentage Spain
pct4gha	float	%9.0g	Scenario 4: Percentage Ghana

The primary variables of interest (i.e., the variables that store the survey participants’ survey answers) are stored in variable names that being with fcase* and fblackcaps*. The fcase1, fcase2, and fcase3 variables correspond to a participant's answer for case 1, case 2, and case 3 of the survey questionnaire. In each case, a participant is asked for ecological compensation in Denmark, Spain, and Ghana. Their answer for each country corresponds to the country after the underscore (i.e., fcase1_denmark is a participant's desire for ecological compensation in Denmark under case 1). The variables fblackcaps* correspond to case 4 and follows the same setup as cases 1 to 3 for the country after the underscore.

**Fig. 1:**
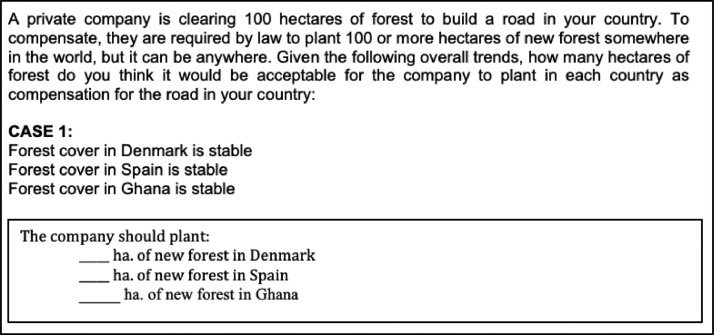
Sample case.

Table 2 provides summary statistics for these main variables of interest. The means represent the average amount participants in our survey desire for ecological compensation. For instance, the variable “fcase1_denmark” means that under case 1 in our survey (see Fig. 1 below for what case 1 is; the rest of the cases can be found in the actual questionnaire), survey participants desire to plant 76.93 hectares of forest in Denmark. The minimum number of hectares of forest a survey participant desires to plan in Denmark is 0 while the maximum number of hectares of forest a survey participant desires to plan in Denmark is 1,000. The rest of the variables in Table 2 are interpreted in a similar way.

**Table 2:** Summary statistics of the main variables of interest.

Variable	Obs	Mean	Std. Dev.	Min	Max
fcase1_denmark	691	76.93	100.61	0	1,000
fcase1_spain	691	72.02	108.99	0	10,00
fcase1_ghana	691	100.64	446.83	0	10,000
fcase2_denmark	691	93.85	163.63	0	2,000
fcase2_spain	691	95.31	212.89	0	3,000
fcase2_ghana	691	106.56	440.76	0	10,000
fcase3_denmark	691	62.91	99.61	0	1,000
fcase3_spain	691	59.29	101.32	0	1,000
fcase3_ghana	691	134.73	319.37	0	5,000
fblackcaps_denmark	691	79.83	145.98	0	1,500
fblackcaps_spain	691	75.53	160.22	0	2,500
fblackcaps_ghana	691	122.09	259.54	0	4,000

*Notes:* Please see Table 1 for the definition of each variable.

The variables case* and pct* are created variables. The variables that start case* sums up a participant's ecological compensation across all countries under case 1 while the variables that start with pct* takes the percent of the sum for a particular country in a particular case. That is, pct1den = fcase1_denmark / case1sum.

## 3 Experimental Design, Materials and Methods

A. Survey Design

The survey design is straightforward. Individuals were presented a general scenario and then asked the amount of forest, in hectares, should be planted in each of our three countries (Denmark, Spain, and Ghana) to compensate for the forest loss in the general scenario (which was envisioned to take place in the country in which they are based). The general scenario that was presented to participants was as follows:

“A private company is clearing 100 hectares of forest to build a road in your country. To compensate, they are required by law to plant 100 or more hectares of new forest somewhere in the world, but it can be anywhere.”

Participants had to write down the amount of forest in hectares under four cases: (1) forest cover in Denmark, Spain, and Ghana is stable, (2) forest cover in one's country is declining while forest cover in the two foreign countries is stable or increasing, (3) forest cover in Denmark and Spain are slightly increasing but forest cover in Ghana is slightly decreasing, and (4) the road causes a decline in blackcaps (a bird), planting a forest in Africa will result in greater benefits for the blackcaps, and forest cover in all countries are stable. A sample of such a case is presented in Fig. 1.

Socio-demographic information, such as age, educational level, civil status, number of children, risk preferences, and various preferences towards Danes, Spaniards, and Ghanaians, were also solicited.

B. Materials

Consent form and survey questionnaire in English are available as supplementary materials of the original research article [1]. The survey was done via paper-and-pen. Participants in Denmark and Spain were seated in tables with partitions while participants in Ghana were seated two to three seats apart. The survey was anonymous, and participants were only known by the ID numbers that were randomly assigned to them. Participants were provided the questionnaire and a pen.

Hardcopies of the survey questionnaire were all brought back to Denmark and separately encoded by two research assistants. The encoded versions were then superimposed against each other as a consistency check. Consistency in encoded data were not incentivized as research assistants were paid by the hour. Any discrepancy in the encoded data were compared to the hardcopy and adjusted as needed.

C. Methods

Our survey participants are university students in Denmark, Spain, and Ghana. Only Danes in Denmark, Spaniards in Spain, and Ghanians in Ghana were allowed to participate in the survey. Participants in Denmark and Spain were recruited through the Online Recruitment of Students for Economic Experiments (ORSEE) [5], a database of participants owned and managed by the University of Copenhagen and the Pompeu Fabra University, respectively, while participants in Ghana were recruited through flyers and in-class announcements. As mentioned above, the survey participants were university students. Therefore, the sample of participants in a country was not representative of its population. Hence, no sampling weights were used. In all countries, participants only interacted with “instructors”, local individuals (i.e., local to the country the survey is taking place at) who were trained together in Denmark to implement the survey but were not part of the research team.

**Fig. 2:**
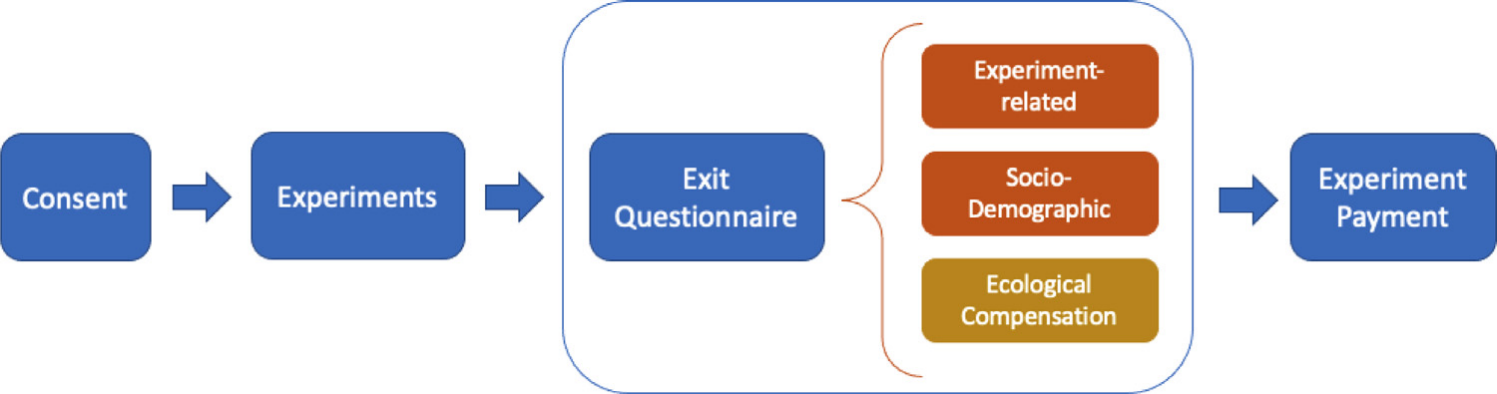
Participant process.

The survey is part of a series of experiments conducted in Denmark, Spain, and Ghana from April to May 2016 [[3], [4], [5], [6], [7]]. Although independent from any of the experiments conducted in terms of research topic, it is given to experiment participants as part of their exit questionnaire. We can see in Fig. 2 where the survey (in brown) fits into the entire process from participant consent to the end of the experiment process. The exit questionnaire given to participants is composed of three categories of questions: those related to the experiments they have participated in, those related to their socio-demographic characteristics, and those related to ecological compensation.

## CRediT authorship contribution statement

**Anna Lou Abatayo:** Conceptualization, Methodology, Data curation, Investigation, Visualization, Validation, Writing – original draft, Writing – review & editing. **Joseph William Bull:** Conceptualization, Methodology, Writing – review & editing. **Niels Strange:** Conceptualization, Methodology, Validation, Writing – review & editing, Supervision.

## Declaration of Competing Interest

The authors declare that they have no known competing financial interests or personal relationships that could have appeared to influence the work reported in this paper.

## Data Availability

Dataset for “Counterintuitive Proposals for Trans-boundary Ecological Compensation Under ‘No Net Loss’ Biodiversity Policy” (Original data) (Mendeley Data).
